# Q Fever and Pneumonia in an Area with a High Livestock Density: A Large Population-Based Study

**DOI:** 10.1371/journal.pone.0038843

**Published:** 2012-06-07

**Authors:** Lidwien A. M. Smit, Femke van der Sman-de Beer, Annemieke W. J. Opstal-van Winden, Mariëtte Hooiveld, Johan Beekhuizen, Inge M. Wouters, Joris Yzermans, Dick Heederik

**Affiliations:** 1 Division Environmental Epidemiology, Institute for Risk Assessment Sciences, Utrecht University, Utrecht, The Netherlands; 2 Netherlands Institute for Health Services Research, Utrecht, The Netherlands; Public Health Agency of Barcelona, Spain

## Abstract

Concerns about public health risks of intensive animal production in the Netherlands continue to rise, in particular related to outbreaks of infectious diseases. The aim was to investigate associations between the presence of farm animals around the home address and Q fever and pneumonia.

Electronic medical record data for the year 2009 of all patients of 27 general practitioners (GPs) in a region with a high density of animal farms were used. Density of farm animals around the home address was calculated using a Geographic Information System. During the study period, a large Q fever outbreak occurred in this region. Associations between farm exposure variables and pneumonia or ‘other infectious disease’, the diagnosis code used by GPs for registration of Q fever, were analyzed in 22,406 children (0–17 y) and 70,142 adults (18–70 y), and adjusted for age and sex. In adults, clear exposure-response relationships between the number of goats within 5 km of the home address and pneumonia and ‘other infectious disease’ were observed. The association with ‘other infectious disease’ was particularly strong, with an OR [95%CI] of 12.03 [8.79–16.46] for the fourth quartile (>17,190 goats) compared with the first quartile (<2,251 goats). The presence of poultry within 1 km was associated with an increased incidence of pneumonia among adults (OR [95%CI] 1.25 [1.06–1.47]).

A high density of goats in a densely populated region was associated with human Q fever. The use of GP records combined with individual exposure estimates using a Geographic Information System is a powerful approach to assess environmental health risks.

## Introduction

Although the number of farms in the Netherlands has been declining for decades, the total number of farm animals is still increasing. Large-scale, intensive animal farming is growing, especially in some specific regions. Concerns about public health risks of increasingly intensive animal production continue to rise, in particular related to emerging zoonotic infectious diseases such as avian and swine influenza, and Q fever [1].

Q fever is caused by *Coxiella burnetii*, an obligate intracellular Gram-negative bacterium. Domestic sheep, goats, and cattle are the most common reservoir, and humans may contract the disease likely by inhalation of contaminated dust and aerosols [2]. Infection may lead to asymptomatic seroconversion in 60% of patients, while a variety of acute clinical manifestations such as flu-like symptoms, hepatitis, and atypical pneumonia may occur in others. Although most acute Q fever patients present with a mild, self-limited febrile illness, some patients have to be hospitalized with a severe pneumonia [3]. Moreover, chronic Q fever may develop in less than 5% of acute cases, manifesting most commonly as endocarditis with a considerable mortality [3]. Protracted fatigue and impaired quality of life are other common sequels in acute Q fever patients [4],[5].

Several community outbreaks of Q fever have been described, often implicating infected domestic ruminants as the source of infection [2], [6]–[10]. *C. burnetii* persists in the environment, and outbreak investigations suggested windborne spread of infective aerosols over several kilometers [6]–[9]. Recently, an outbreak of unprecedented scale occurred in the Netherlands, with more than 3,500 patients registered by regional public health services between 2007 and 2009, and a hospitalization rate of 20% [11], which is remarkably higher than the 2 to 5% that are reported in earlier literature [3]. Single dairy goat farms with high abortion rates, so-called abortion storms, due to *C. burnetii* infections were implicated as the most likely source in a few studies that investigated local clusters [1], [7]. In 2010, the number of cases started to decrease, likely as a result of intervention measures including culling of pregnant goats and sheep on Q fever positive farms, and mandatory vaccination of dairy goats and dairy sheep implemented in 2010 [1], [12]. It has been suggested that the increased goat density over recent years has probably contributed to the unparalleled scale of the human Q fever outbreak in the Netherlands [1], but this relationship has thus far not been studied.

We carried out a study investigating respiratory health effects among individuals living in the vicinity of animal farms in a highly populated area in the south of the Netherlands, using general practitioners' (GP) medical records for the year 2009 of more than 100,000 patients living in this area [13]. The study area and period coincided with a high incidence of Q fever. The present study is the first to investigate associations between the presence of farm animals around the home address and GP-registered Q fever and pneumonia. Given the implication of goat farms as the most likely source, we focused the analyses on goat exposures, and investigated the role of other farm animals as additional risk factors or potential confounders.

## Methods

### Ethics Statement

The study was carried out according to Dutch legislation on privacy and the Code of Conduct for Medical Research [14]. Patients' privacy was ensured by keeping medical information and address records separated at all times, by using a Trusted Third Party. According to Dutch legislation, medical ethical approval was not required for this research.

### Study population

In the Dutch health care system, citizens are on the list of just one GP, who acts as a gatekeeper to secondary care. Dutch GPs keep electronic medical records (EMR) in which morbidity data of the patients are registered. General practices outside the larger cities in the eastern part of the province of Noord-Brabant and the northern part of the province of Limburg, a region with a high density of farm animals, were requested to participate. Practices were only included in the study if they met pre-defined registration quality criteria: 1) practices had to record diagnostic information in the patients' EMR using the International Classification of Primary Care (ICPC) [15]; 2) an ICPC code had to be assigned to at least 50% of the morbidity records in the EMR; and 3) practices had to record during at least six months of the year. Twenty-seven practices met these criteria and were included in the study. Participating practices were located in small towns and villages with a population of less than 25,000. Data were collected from EMR of all 105,870 enlisted patients for the year 2009. This includes patients who did not actually consult their GP in 2009. Because the study was focused on neighboring residents of livestock farms [13], we excluded 3,942 patients (3.7%) who had a high likelihood to be living on a farm (distance between home address and animal stables <50 m). Analyses were carried out in all 92,548 patients aged 70 years or younger: 22,406 children (0–17 y) and 70,142 adults (18–70 y).

### Data collection

Morbidity data were derived from the EMR and from all prescriptions issued by the GPs. Consultations concerning the same health problem were clustered into episodes of care defined as all encounters for the management of the same specific health problem. Episodes were constructed using EPICON, a computerized algorithm that groups ICPC-coded contact records from EMR into episodes of care [16]. In the Netherlands, Q fever is registered by GPs under the ICPC code ‘other infectious disease’ (A78). Despite the broad name, ‘other infectious disease’ is normally only used for patients with Q fever or Lyme disease. The ICPC code for pneumonia is R81.

### Farm animal density around the home address

**Figure 1:**
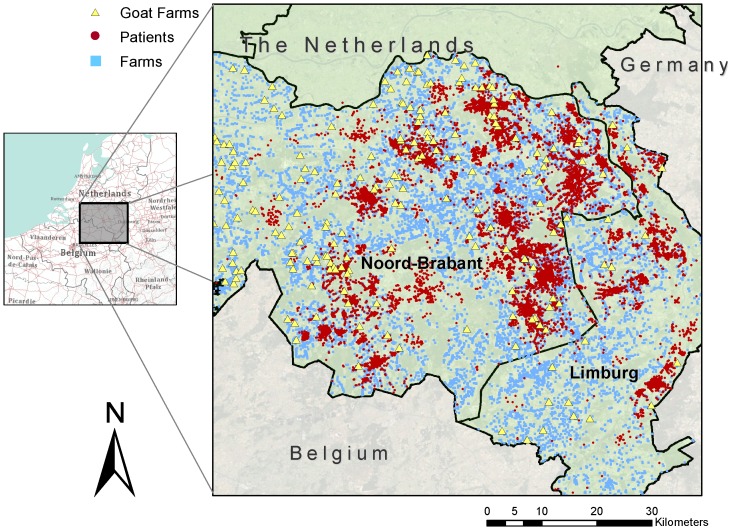
Study area: the eastern part of the province of Noord-Brabant and the northern part of the province of Limburg. Dots represent residential addresses of 92,548 study subjects. Squares represent farms holding a licence to keep livestock. Triangles represent goat farms.

The precise coordinates of all animal farms in the study area, and the type and number of animals were obtained from the provincial database of mandatory environmental licences for keeping livestock in 2009. Patients' residential addresses were geocoded, and distances between the home address and all animal farms within a 1 km radius were calculated using a geographic information system (ArcGis 9.3.1, Esri, Redlands, CA). Binary variables indicating the presence of a specific type of farm animal within 1 km from the home address were created. In addition, all goat farms (farms keeping goats as the main type of animal, or other livestock farms with at least 50 goats) within 5 km from the home address were identified. The shortest distance between a goat farm and home address, and the total number of goats within 5 km were computed. Figure 1 shows a map of the study area, indicating the presence of livestock farms around subjects' homes. In total, there were 180 registered goat farms in this area with an average (permitted) number of 1,307 goats (sd 1,195).

### Statistical analysis

Univariate tests of association between patients' characteristics, farm animal exposure variables, and pneumonia and ‘other infectious disease’ were performed using Chi-square test or t-test. The shape of the association between goat exposure (number of goats within 5 km and distance to nearest goat farm) and disease were investigated by means of generalized additive modelling (smoothing) as described earlier [17]. In addition, associations between the number of goats and health outcomes were analyzed by means of multiple logistic regression analysis, with adjustment for age, sex, and the presence of other farm animals, showing mutually adjusted effects of the number of goats and the presence of other animals than goats around the home address. In the multiple logistic regression models, the number of goats was categorized into four quartiles based on an equal number of cases in each category, which provides a similar variance for odds ratios across categories [18]. Standardized household income data, a proxy for socioeconomic status (SES), were obtained from Statistics Netherlands for 84.9% of the study population and linked to the GP data. For privacy protection reasons, analyses using SES information had to be completed on-site at Statistics Netherlands, and were therefore limited to some of the key associations. Results are shown without adjustment for income, because the ICPC codes under study were not associated with household income (P>0.05) and corrections for household income did not alter results.

## Results

### Characteristics of patients

In total, 702 adults and 221 children were diagnosed with pneumonia, and 470 adults and 52 children received a diagnosis of ‘other infectious disease’ (Table 1). Univariate tests of association showed that adult patients with pneumonia were older than subjects who were not diagnosed with pneumonia or ‘other infectious disease’ in 2009 (control subjects). A shorter distance between the home address and the nearest goat farm, a higher number of goats within a 5 km radius, and the presence of poultry within a 1 km radius were also associated with pneumonia. Adult patients with ‘other infectious disease’ were more often male, were older, lived closer to goat farms, had a higher number of goats around their home, and lived more often in the vicinity of swine, cattle and sheep than control subjects. Conversely, adult patients with ‘other infectious disease’ lived less often in the vicinity of mink farms. Among children, the presence of goats around the home address was also associated with a diagnosis of ‘other infectious disease’, whereas goat or other farm animal exposures were not associated with pneumonia in children.

**Table 1:** Characteristics of patients diagnosed with pneumonia or ‘other infectious disease’ and control subjects.

	Adults			Children		
Characteristic	Control subjects	Pneumonia	‘Other infectious disease’†	Control subjects	Pneumonia	‘Other infectious disease’†
n	68,989	702	470	22,134	221	52
Male gender (%)	51.3	54.3	56.8*	51.4	56.1	57.7
Age (years, mean ± sd)	44.8±14.4	51.1±13.6**	47.3±13.2**	8.9±5.1	6.3±4.9**	9.4±5.3
Presence of farm animals within 1 km (%)						
Swine	82.4	81.8	89.4**	84.0	87.8	84.6
Poultry	53.5	58.6*	51.1	56.3	59.3	57.7
Cattle	87.5	87.0	94.7*	89.5	90.1	94.2
Goat	12.5	15.7*	21.5**	14.1	18.1	26.9*
Sheep	45.9	46.0	52.1*	48.2	50.7	51.9
Mink	7.3	5.7	3.0**	8.1	6.3	7.7
Distance to nearest goat farm (km, mean ± sd)	2.58±1.33	2.40±1.32**	1.91±1.12**	2.53±1.32	2.46±1.43	1.99±1.11*
Number of goats within 5 km (×1000, mean ± sd)	6.45±6.12	7.45±6.49**	13.02±7.05**	6.70±6.28	7.31±6.47	8.54±6.60*

† Nineteen adults and one child received a diagnosis pneumonia *and* ‘other infectious disease’ in 2009; ‘other infectious disease’ is the diagnosis code used by GPs for registration of suspected Q fever.

* P<0.05, Chi-square test or t-test.

** P<0.001, Chi-square test or t-test.

### Association between goat exposure and pneumonia and ‘other infectious disease’

**Figure 2:**
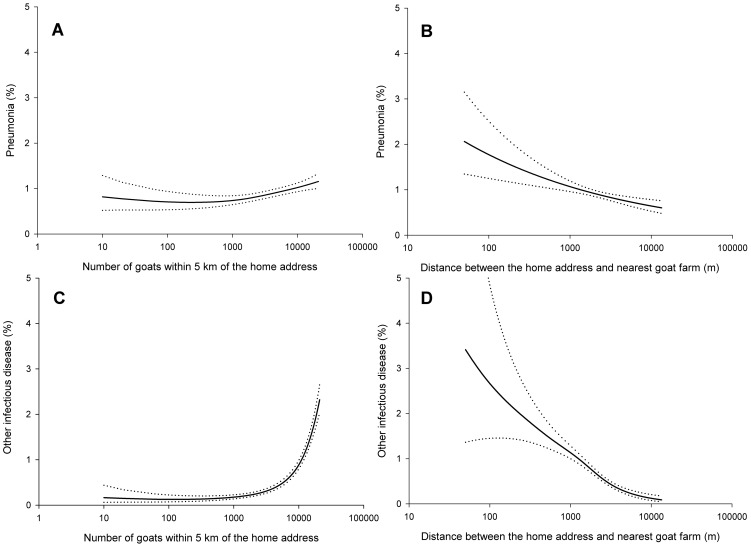
Smoothed plots with 95% confidence bands representing associations of the number of goats within 5 km around the home address with pneumonia (A; P = 0.001) and ‘other infectious disease’ (C; P<0.0001), and associations of distance to nearest goat farm with pneumonia (B; P = 0.0002) and ‘other infectious disease’ (D; P<0.0001) among 70,142 adults. Associations were adjusted for age and sex.

The shape of the associations between distance to the nearest goat farm and number of goats around the home address and the Q fever-related outcomes in adults are shown as smoothed plots (Figure 2). Clear trends between a smaller distance to the nearest goat farm, a higher number of goats around the home address, and a higher disease incidence were observed, and all associations were statistically significant (linear component P≤0.001). A steep, non-linear increase of ‘other infectious disease’ with an increasing number of goats around the home address was found, showing a predicted incidence of 0.1% in adults with 100 goats within 5 km, 0.9% in adults with 10,000 goats within 5 km, and 2.2% in adults with 20,000 goats within 5 km of the home address (Figure 2C). The predicted incidence of ‘other infectious disease’ was 0.1% in those living at 10 km from the nearest goat farm, 1.1% in adults living at 1000 m, and 3.4% among adults living at 50 m from a goat farm (Figure 2D). When these two goat exposures were included as determinants in one model, only the number of goats remained a statistically significant risk factor (P<0.0001), whereas the distance to the nearest goat farm was no independent determinant (P = 0.40).

In children, positive and statistically significant exposure-response relations were found for goat exposures and ‘other infectious disease’ (P<0.05), but not for pneumonia.

### Association of goat and other farm animal exposure with pneumonia and ‘other infectious disease’

Table 2 shows that estimates for association between the number of goats and Q fever-related outcomes in adults were also statistically significant when adjusted for other farm animals. The association with ‘other infectious disease’ showed a particularly strong exposure-response trend, with an OR [95%CI] of 12.03 [8.79–16.46] for the fourth quartile (>17,190 goats) compared with the first quartile (<2,251 goats). The presence of sheep within 1 km from the home address showed a negative association with ‘other infectious disease’ (OR [95%CI] 0.72 [0.58–0.89]), whereas the presence of poultry was associated with an increased risk of pneumonia (OR [95%CI] 1.25 [1.06–1.47]). The latter associations were all adjusted for presence of all other farm animals and the number of goats. The positive associations between the presence of swine farms and cattle farms and ‘other infectious disease’, and the negative association between the presence of mink farms and ‘other infectious disease’ (Table 1, univariate models) were not statistically significant after correction for goat and other farm animal exposures (Table 2).

**Table 2:** Risk factors for pneumonia, and ‘other infectious disease’ in 70,142 adults.

Determinant	Pneumonia	‘Other infectious disease’†
	OR (95% CI)	OR (95% CI)
Male gender	1.13 (0.97–1.31)	1.25 (1.04–1.50)
Age (per 10 years)	1.39 (1.31–1.47)	1.13 (1.06–1.20)
Number of goats within 5 km		
0–2,250	1.00 (Reference)	1.00 (Reference)
2,251–7,250	1.45 (1.20–1.76)	1.98 (1.42–2.75)
7,251–17,190	1.34 (1.10–1.64)	4.05 (3.01–5.46)
17,191–20,960	1.68 (1.28–2.21)	12.03 (8.79–16.46)
Presence of farm animals within 1 km		
Swine	0.96 (0.77–1.21)	1.10 (0.78–1.56)
Poultry	1.25 (1.06–1.47)	0.88 (0.73–1.07)
Cattle	0.90 (0.70–1.16)	1.57 (0.99–2.50)
Sheep	0.93 (0.79–1.09)	0.72 (0.58–0.89)
Mink	0.89 (0.64–1.23)	0.72 (0.42–1.24)

Odds ratios and 95% CI were adjusted for all variables in the Table using multiple logistic regression analysis.

† ‘Other infectious disease’ is the diagnosis code used by GPs for registration of suspected Q fever.

## Discussion

We conducted a large population-based study of GP patients in the south of the Netherlands, which coincided with a large Q fever outbreak in this region. We found that a large number of goats around the home address and a short distance to the nearest goat farm were associated with an increased risk of ‘other infectious disease’, which was used by GPs to register Q fever, and pneumonia. A significantly increased incidence of pneumonia was also observed among adults living within 1 km of one or more poultry farms.

Q fever is usually considered to be a rare and mainly occupational disease in farmers, abattoir workers, and veterinarians, although community outbreaks around farms with infected ruminants, especially during the kidding season, are not unusual [2]. Since 2007, a major Q fever outbreak occurred in the south of the Netherlands. More than 2,300 cases were notified nationwide in 2009, the year the data of the present study originate from [11]. Two recent epidemiological studies have linked single *C. burnetii* positive dairy goat farms to clusters of human Q fever [7], [19]. In 2007, a cluster of 55 notified cases in a rural village was investigated. Living close to ruminant farms, including a large dairy goat farm with abortion waves due to *C. burnetii*, was identified as a risk factor [19]. An urban cluster of 96 cases in 2008 was clearly connected to a Q fever positive goat farm. People living within 2 km of this farm had a strongly increased risk of Q fever compared with those living more than 5 km away [7]. A retrospective study of hospitalizations for lower respiratory tract infections concluded that Q fever clusters related to infected ruminant farms probably preceded the 2007 outbreak [20]. A study investigating the effect of environmental conditions around infected goat and sheep farms on Q fever transmission to humans suggested that vegetation density, groundwater conditions, and cattle density in a 5 km radius around infected farms were associated with transmission, whereas goat density in a 5 km radius around infected farms was not an independent risk factor [21]. However, goat and cattle density around patients' residential addresses were not considered in these analyses [21]. In the present study, the presence of cattle within 1 km of the home address was positively but not significantly associated with ‘other infectious disease’. Although *C. burnetii* is widespread in Dutch dairy cow herds [22], it is not clear to what extent cattle may contribute to the transmission of Q fever to humans.

An outbreak of 147 Q fever cases in the UK in 1989 coincided with a large increase in the total number of sheep during the previous two years [6]. In recent years, there has been a sharp increase in the number of dairy goat farms in the Netherlands, in particular in the densely populated province of Noord-Brabant [1]. Between 2000 and 2009, the total number of dairy goats more than doubled to almost 375,000 [1]. It has been hypothesized that the remarkable increase in goat density and extension of farms over recent years may have contributed to the unparalleled scale of the human Q fever outbreak in the Netherlands [1]. The present study supports this hypothesis by demonstrating a clear exposure-response relationship between the number of goats within a 5 km radius of the home address and Q fever-related outcomes. The presence of sheep within 1 km of the home address was negatively associated with ‘other infectious disease’ after adjustment for the number of goats. Q fever abortions were mainly diagnosed on dairy goat farms, and only on a few dairy sheep farms [1]. The database that we used did not distinguish between goats and sheep kept for dairy production or other purposes. However, in the Netherlands, most goats are kept on dairy goat farms, while most sheep are kept for other purposes.

The observed increased risk of pneumonia among patients living in the vicinity of poultry is less easily explained. Chickens and other poultry may be carriers of *C. burnetii*, but significant transmission to humans is unlikely, and has not been described [2]. Individuals living near poultry farms may be exposed to other pathogens such as influenza viruses and to increased levels of air pollutants such as particulate matter and endotoxin [23]. Prolonged exposure to fine particulate matter might predispose individuals to hospitalization with community-acquired pneumonia, as suggested in a study among older Canadians [24]. In animal studies, exposure to ambient particulate matter compromised host ability to handle ongoing pneumococcal infections [25]. Very little information exists about exposures and respiratory health effects among neighbors of (poultry) farms [26]. There is some evidence of adverse effects on lung function in populations living near intensive livestock and poultry farms [27], [28], but it is unclear whether such effects may underlie increased susceptibility to pneumonia. Although we attempted to adjust optimally for other farm animal exposures, we cannot exclude the possibility of residual confounding by goat exposure. It would therefore be interesting to investigate whether associations of poultry farm exposures with pneumonia would also be observed in a period with no Q fever outbreak.

Our study was the first to investigate environmental risk factors for Q fever by linking GP medical records with farms around patients' residential addresses. This approach has strengths and limitations. A strength of our study was the availability of precise residential addresses, which we geocoded and linked to livestock registrations. Thus, for each of the 92,548 patients included in the analyses, livestock farming activities around the home were assessed on an individual basis. We used farm license data, which may overestimate the number of animals actually present at a facility. It was not possible to trace goat and sheep farms that were *C. burnetii* positive at the time of our study, because mandatory monitoring of bulk tank milk samples for *C. burnetii* started in October 2009. Data on farm locations with abortion storms are registered by the Animal Health Service, but these data were not systematically collected and were not available for our research. Despite limitations of the exposure assessment, we observed strong and biologically plausible associations between livestock farming activities around patients' homes and GP-registered Q fever and pneumonia.

In the Netherlands, every citizen is obliged to be on the list of just one general practice. Because we used routinely collected records from all patients from 27 rural GP practices, our study has a major strength, namely the lack of selection bias and recall bias. A drawback of using GP records is the limited number of potential confounders available. We only adjusted risk estimates for age and gender. As expected [3], male gender and older age were associated with ‘other infectious disease’. Symptomatic Q fever is known to occur less often in children [3], which is also in accordance with our findings. Socio-economic status (household income) was not associated with Q fever outcomes. We did not have information about childhood farm exposures, which may be associated with protective immunity, but patients who were likely to live on a livestock farm were excluded from the analyses.

A limitation of our study was the lack of laboratory confirmation of Q fever. In the Netherlands, notification criteria for a confirmed case are fever, pneumonia, or hepatitis, combined with the detection of antibodies to *C. burnetii* [11]. Dutch GPs register Q fever under the ICPC code ‘other infectious disease’. However, the same code is also used for patients with Lyme disease. In the current study, the incidence of ‘other infectious disease’ was 6.8 per 1000 adults and 2.3 per 1000 children. In 2006, the year before the outbreak, the nationwide incidence of ‘other infectious disease’ was 2.5 per 1000 patients and 2.3 in the study area, according to data obtained from a national GP network. In 2009, the incidence rates were 2.2 and 3.3, thus a small decrease in the nationwide practices, but a substantial increase in the study area [13], [29]. Data about the number of Lyme disease patients in the Netherlands are unavailable, but a recent Dutch study showed that the incidence of GP consultations because of erythema migrans, a specific symptom of Lyme disease, was 1.3 per 1000 in 2009 [30]. We also studied pneumonia as a potential Q fever-related outcome, because pneumonia was the diagnosis made most frequently among the notified Q fever patients in the epidemic in the Netherlands (61.5%), whereas endocarditis (3.1%) and hepatitis (0.4%) were relatively rare [31]. However, pneumonia cases unrelated to *C. burnetii* could not be distinguished. Thus, due to the design of our study, a certain degree of misclassification of Q fever status could not be avoided. However, this misclassification of disease is probably not related to exposure status. During our study period, residents were not yet informed of the presence of tank milk positive farms within a 5 km radius of their homes (this information started to be made public in December 2009). Although patients and GPs are aware that they are living in a region with a high density of livestock farms, it is not likely that they can precisely estimate the farm animal density around patients' homes. In a German study, a very low level of agreement (17%) has been shown between self-reported number of livestock farms within 500 m of subjects' home address and the actual number of farms. A similar lack of awareness can be assumed in the Netherlands, leading to non-differential misclassification of disease and underestimation of the effect of farm exposures.

In conclusion, this study has shown a clear exposure-response relationship between the number of goats within a 5 km radius of the home address and GP-registered Q fever and pneumonia during a major outbreak of human Q fever. Our findings strongly support the hypothesis that the high density of goats in the south of the Netherlands contributed to the outbreak. We also observed an increased risk of pneumonia among patients living in the vicinity of poultry which may be explained by exposure to other pathogens or increased levels of air pollutants. The use of GP records in combination with individual estimates of exposure using a Geographic Information System is a powerful approach to assess environmental health risks.
